# Stereological estimation of mean nuclear volume as a prognostic factor in canine subcutaneous mast cell tumors

**DOI:** 10.1177/03009858251315094

**Published:** 2025-02-19

**Authors:** José Catarino, Ana Macara, André Barros, David Ramilo, Filipa Coelho, Joana Santos, Pedro Faísca

**Affiliations:** 1 Faculdade de Medicina Veterinária, Universidade Lusófona – Centro Universitário de Lisboa, Lisbon, Portugal; 2 DNAtech, Lisbon, Portugal; 3 Instituto Politécnico da Lusofonia, Lisbon, Portugal; 4 Comprehensive Health Research Centre, Universidade de Évora, Évora, Portugal; 5 Instituto Gulbenkian de Ciência, Oeiras, Portugal

**Keywords:** dogs, mean nuclear volume, prognosis, stereology, subcutaneous mast cell tumor, surgical pathology

## Abstract

Classification schemes regarding canine subcutaneous mast cell tumors (csMCTs) remain elusive, lack consensus, and are prone to interobserver variability and bias. This observational study aimed to assess the reproducibility and the prognostic significance of volume-weighted mean nuclear volume ( $\bar{v_{v}}$ ), a stereological estimation offering insights into nuclear size and its variability, in csMCTs. Thirty csMCTs were selected with information regarding outcome, and $\bar{v_{v}}$ was estimated using the “point-sampled intercept” method. Interobserver and intraobserver $\bar{v_{v}}$ reproducibility yielded concordance coefficients near or above 0.90. Regarding previously reported risk factors (pattern, mitotic count, and multinucleated cells), no statistically significant differences were identified between patterns and clinical outcome, nor between patterns and $\bar{v_{v}}$ ; however, the infiltrative pattern was represented more in the poorer outcome group and had higher $\bar{v_{v}}$ values. When comparing $\bar{v_{v}}$ and clinical outcome, a statistically significant difference emerged. Cases with poorer outcomes had higher $\bar{v_{v}}$ values ( $\tilde{x}$ = 192.9) than cases with more favorable outcomes ( $\tilde{x}$ = 120.5), and this association was statistically significant on both univariable and multivariable analyses. This study suggests that $\bar{v_{v}}$ is highly reproducible and is associated with clinical outcome in csMCTs.

Mast cell tumors (MCTs) are the most common skin neoplasia in dogs. The diagnosis is usually simple and straightforward; however, their biological behavior is unpredictable.^3,16^ In the skin, they are classified as cutaneous, when the neoplasm affects the dermis and can infiltrate either the epidermis or the subcutaneous tissue,^17,28^ or subcutaneous, when the tumor is totally surrounded by subcutaneous adipose tissue.^34,35^ Grading remains the primary means of determining the prognosis for canine cutaneous mast cell tumors (ccMCTs),^9,22,28,36^ with 2 validated and commonly used systems: the 3- and the 2-tier grading systems.^17,25^

Newman et al^23^ and Thompson et al^34^ suggested that MCTs affecting only the subcutaneous tissue should not be graded the same way as cutaneous tumors and in 2011, Thompson et al assessed a set of parameters, 3 of which were found to have prognostic value: pattern of growth, mitotic count (MC), and multinucleated cells. The growth pattern was classified into 3 categories based on the sub-macroscopic appearance of the tumor: circumscribed, combined, and infiltrative, with the latter identified as a significant risk factor associated with decreased survival time. For multinucleated cells and MC, tumors that have multinucleated cells and/or an MC > 4 were also associated with a poorer prognosis. These parameters can be regarded as difficult to evaluate, often leading to interobserver variation and bias.^4^ The MC has recently become a subject of intense debate, with several papers highlighting the importance of standardizing the methodology for MC assessment, such as specifying regions for mitotic counting, determining the overall area for accurate assessment, and establishing standardized criteria for the morphological characteristics of a mitotic figure.^10,12,19^ Threshold values identified in the literature for MC can sometimes conflict,^34,37^ further complicating the application of this parameter. More recently, the development of deep-learning algorithms capable of detecting mitotic figures in digital whole-slide images has further shown that manual mitotic count is strongly dependent on the area selected.^1,4^ Similar criticisms can be applied to the assessment of multinucleated cells. In addition, a recent study concluded that multinucleation of canine subcutaneous MCTs (csMCTs) was not associated with a poorer outcome.^14^ In comparison with ccMCTs, there are very few studies concentrating on prognostic parameters of csMCTs. This can partially be explained by the perception that csMCTs are tumors with low incidences of local recurrence and distant metastasis, and higher survival times when compared with ccMCTs.^23,34^ Nonetheless, recent research suggests otherwise, with Cherzan et al^9^ reporting that among 45 cases of csMCTs, 17.7% experienced local recurrences and 26.7% experienced lymph nodes metastases. In 2021, a consensus paper on MCTs recommended using the 2-tier grading system for all MCTs.^37^ This was further validated in 2024 by Sabatini et al^28^ demonstrating its ability to identify ccMCTs and csMCTs with aggressive behavior, regardless of their growth model.

Recently, the prognostic value of volume-weighted mean nuclear volume ( $\bar{v_{v}})$ ), was explored in ccMCTs.^7^ Stereology is considered the gold standard for estimating quantitative properties of 3-dimensional objects in histological samples.^5,11^ These methods rely on statistical sampling and geometrical principles to extract 3-dimensional information from 2-dimensional sections, minimizing assumptions about an object’s shape and orientation, and enabling accurate and consistent measurements of various parameters, such as nuclear size.^5,7^ The most effective approach for assessing nuclear size through stereological methods is the “point-sampled intercept” method.^7,15,21^ This method calculates $\bar{v_{v}}$ , where larger nuclei have higher probabilities of being sampled.^7,21^ As a result, this assessment reflects both the size and variation in nuclear shape, increasing not just as individual nuclei grow larger but also with increased diversity in nuclear sizes. Therefore, $\bar{v_{v}}$ can be considered a quantitative assessment of nuclear pleomorphism, nuclear size, and its variability.

The present observational study aimed to estimate $\bar{v_{v}}$ on csMCTs to evaluate its reproducibility between and within operators and assess its prognostic value.

## Materials and Methods

### Case Selection and Outcome

All csMCTs diagnosed between 2019 and 2022 were selected for this study from the archives of DNATech Laboratory, Lisbon, Portugal. To be included in the study, the following inclusion criteria had to be met: the dog had to be treated by surgical excision alone and the diagnosis had to be made at least 2 years before the start of the study or if death related to the tumor was confirmed. Cases were also selected based on the availability of complete data regarding clinical follow-up, as well as age, sex, breed, and surgical margins. Referring veterinarians were contacted by email or phone, and clinical follow-up data regarding the existence of postsurgical local recurrence, metastasis, and/or MCT-related death were collected. Recurrence or metastasis had to be confirmed either by histology or suspected via clinical exam and/or sonography (Supplemental Table S1).

Dogs with postsurgical resolution of disease, with a minimum follow-up period of 2 years, were given an outcome value of 0 (OC0), whereas outcome value of 1 (OC1) included cases that died or were euthanized as a result of disease progression (ie, local recurrence or development of nodal or visceral metastasis). The lateral and deep surgical margins (cm) were measured on formalin-fixed biopsies by a histology technician, reviewed by a pathologist, and then compared across groups.

Cases that were lost to follow-up or in which the interviews were not able to provide complete clinical data were not included in the study. Out of an initial pool of 104 cases, only 30 met the inclusion criteria and were thus incorporated into the study. Subsequently, the initial diagnoses were independently confirmed by 2 different pathologists (PF—PhD, Professor of Veterinary Pathology with more than 20 years of experience and JC—MSc, Professor of Veterinary Pathology with 6 years of experience).

Three–micrometer-thick sections (1 section per tumor) were collected from the same paraffin block used for diagnosis. The tumor had been previously trimmed by sectioning it perpendicular to its longest axis, and the slab with the largest surface area was selected for further processing. Each section was routinely processed and stained with hematoxylin and eosin, and each tumor was classified as described by Thompson’s et al.^34^ Histological patterns were characterized as either circumscribed, infiltrative, or combined. The MC was defined as the number of mitotic figures/2.37 mm^2^. The entire slide was initially screened, and MCs were performed in tumor regions with greatest mitotic activity, avoiding poorly cellular, edematous, and inflamed areas. Multinucleation (more than 1 nucleus) was recorded as present if there was at least 1 multinucleated cell in 2.37mm^2^.^34^

### Stereology

**Figure 1.**
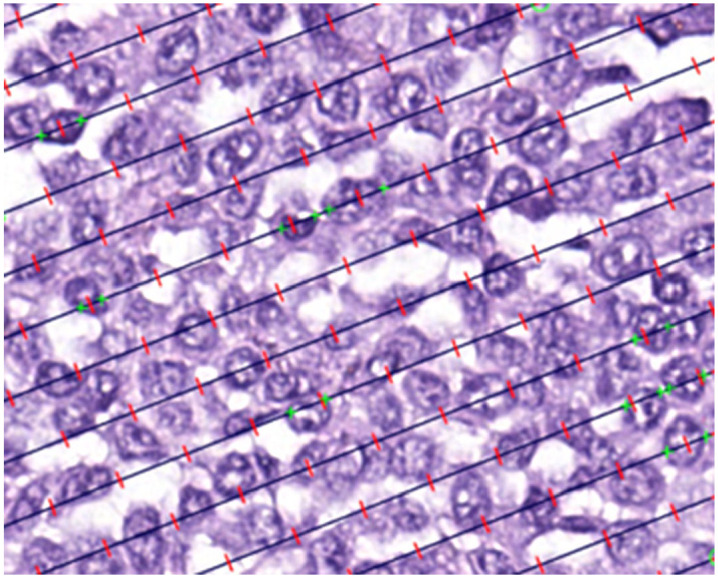
Measuring mean nuclear volume is achieved through the point-sampled intercept method. Fields of view within a mast cell tumor are automatically produced using a consistent step size. Points (represented by red hash marks) sample the nuclear profiles, and the associated test-lines generate linear intercepts across these profiles. To trace the length of the intercepts, the nuclear borders are marked in the line’s direction (depicted by green hash marks). The goal is to measure ~75 intercepts per tumor.

$\bar{v_{v}}$ was estimated as previously described by Casanova et al^7^ on cutaneous MCTs. Measurements were performed on newCAST stereological software (Visiopharm) after obtaining whole-slide images, using the NanoZoomer-SQ Digital slide scanner (Hamamatsu Photonics) at 800× magnification, with a resolution of 20.291 pixels/um^2^, equivalent to a pixel size of 0.222 × 0.222 μm. In each slide, the whole tumor region was delineated manually at low magnification and fields of view (1000X, A = 10,300.84 µm^2^) were automatically selected by the software in a systematic and random manner. According to Gundersen and Jensen, approximately 75 nuclei per tumor are needed to accurately estimate $\bar{v_{v}}$ .^15^ The number of fields of view required to measure the appropriate number of nuclei was influenced by the tumor’s cellularity. Fields where nuclear borders were poorly distinguishable were excluded from measurement. $\bar{v_{v}}$ of csMCTs was estimated according to the point-sampled intercept method in which nuclei are sampled with probes of test-lines and associated points added onto the fields of view. Each sampling field featured parallel lines with a known length, and every time a nucleus was hit by a point, the associated test-line created an intercept across the nuclear profile, whose length was measured in micrometers by marking the nuclear borders (Fig. 1). Lines were randomly rotated between fields, allowing randomization of sampled nuclei and the orientation in which these were sampled and measured. The set of nuclear measurements were introduced into the formula $\bar{v_{v}}$ = $\frac{\pi }{3n}\sum_{i=1}^{n}l_{0}^{3}$ , inwhich, $\bar{v_{v}}$ represents the weighted mean nuclear volume of all measured nuclei. The length of each intersection $\left(l_{0}\right)$ was raised to the power of 3 ( $l_{0}^{3}$ ), converting the measurements to cubic micrometers (μm^3^), and *n* was the number of measured intersections. The analyses were completed in duplicate by 2 separate observers, for a total of 4 independent analyses (JC, 2 analyses; AM, 2 analyses). Each observer performed the duplicate analyses separately, while blinded to clinical outcomes. The interobserver washout period was 1 month, while intraobserver assessments were conducted on the same day. The multilayer randomization, involving fields of view, cells, and line orientation for nuclear measurements, made it impossible to select the same fields, cells, and line orientations even when the assessments were performed successively.

### Descriptive Statistics

Statistical analysis was performed using Microsoft Excel and IBM SPSS Statistics, Version 29.0 (Armonk, New York). Descriptive statistics were calculated for both categorical and quantitative variables. For categorical variables, such as sex and breed, frequencies and percentages were determined and reported. For quantitative variables, such as age and $\bar{v_{v}}$ , the mean and standard deviation (SD) were calculated, along with the quartiles (Q1, median, and Q3) and ranges.

### Statistical Assessment of Reproducibility of $\bar{v_{v}}$

Normality was assessed using the Shapiro-Wilk test, and a parametric or nonparametric approach was selected accordingly. To access $\bar{v_{v}}$ reproducibility, each independent analysis (2 analyses each by 2 separate observers) was grouped into a set of 30 $\bar{v_{v}}$ values, with sets labeled as C1 through C4. The analyses were conducted by 2 observers (C1–C2, C3–C4), who calculated $\bar{v_{v}}$ twice for all tumors. The differences between measurements within sets and between each set of measurements and the average were assessed using the nonparametric Friedman test. Lin’s concordance correlation coefficient was performed to access the agreement between each pair of observers and between each observer, and the average of the 4 measurements.

### Statistical Assessment of Prognostic Value of $\bar{v_{v}}$

The Shapiro-Wilk test was used to assess normality, and based on the results, either a parametric or nonparametric approach was chosen. Before any model testing, independence of observations was assured. To confirm the assumptions for the linear regression model, the linearity was evaluated visually by plotting the continuous variables (age and mean nuclear volume) against the log odds of the outcome, overlaid with an LOWESS curve to check for linear patterns. Homoscedasticity was evaluated using graphical methods, by examining residual plots to ensure that the variance of residuals was constant across all levels of the independent variables. The normality of residuals was assessed using probability-probability (P-P) plots. In the probability-probability plots, the observed cumulative probabilities of the residuals were compared against the expected cumulative probabilities under a normal distribution. Residuals following the 45-degree reference line indicated adherence to the normality assumption. Multicollinearity was assessed using Spearman’s (ρ) and Pearson’s (*r*) correlation coefficients, considering values above 0.7 as indicative of multicollinearity. To evaluate outliers, the Mahalanobis distance was assessed.

For the logistic regression models, assumptions were confirmed similarly. The linear relationship between continuous variables and the log odds of the outcome was evaluated visually, as previously mentioned. Multicollinearity was assessed similarly to the linear regression model, and outliers were also identified using Mahalanobis distance. The overall goodness-of-fit of the logistic regression models was evaluated using the Hosmer-Lemeshow test, with a *P*-value greater than.05 indicating an acceptable fit. McFadden’s pseudo-R^2^ was used to assess the explanatory power of the models, indicating how well the model explained the variability in the outcome. The independent variables included $\bar{v_{v}}$ , pattern, age, sex, breed, margins, and MC, while the dependent variable was the outcome. Due to the large breed dispersion, breeds were dichotomized and categorized as either purebred or mixed breed. The MC was analyzed using 2 different approaches: first as a continuous variable and second by dichotomizing it into 2 groups: MC = 0 and MC > 0. Univariate logistic regressions were conducted to evaluate relationships between clinicopathological characteristics and the outcome. A multivariate logistic regression, including age and sex was performed to examine the relationship between patterns and the outcome while the relationship between patterns and $\bar{v_{v}}$ was assessed using the Kruskal-Wallis test. A linear regression was performed to evaluate the association between age and mean nuclear volume $\bar{v_{v}}$ .

Finally, to assess the relationship between $\bar{v_{v}}$ and the outcome, a multivariable logistic regression model was fitted while examining the impact of potential demographic confounding variables (ie, age, sex, and breed). For a variable to remain in the final model, it had to be statistically significant (ie, a significance level of 5%) or act as a confounding variable. A confounding variable was defined as a nonintervening variable (ie, not in the causal pathway between $\bar{v_{v}}$ and clinical outcome) whose removal from the model results in a 20% change in the model coefficient for mean nuclear volume. Interaction effects were not examined due to the limited sample size.

For linear and logistic models, coefficients and odds ratios (ORs) were reported, respectively, with their 95% confidence intervals (95% CIs) and *P*-values. A significance level of 5% was used for all statistical analyses (ie, alpha = .05).

## Results

### Cohort Description

Thirty csMCTs were selected. Fourteen csMCTs (47%) were diagnosed as infiltrative, 7 (23%) as combined, and 9 (30%) as circumscribed (Table 1). Only 3 tumors (10%) had multinucleated cells (Table 1), and due to the small number of cases inferential analysis was not performed. The average MC was 1.5 (SD = 4.9), with a median of 0 (Table 2). The MC was then divided into 2 groups: MC = 0 (25 cases) and MC > 0 (5 cases) (Table 1).

**Table 1.** Descriptive statistics for categorical variables of patient demographics and tumor characteristics for all cases and by outcome status.

	Pattern			Multinucleation		Mitotic Count (Dichotomized)		Sex		Breed	
	Infiltrative	Combined	Circumscribed	Present	Absent	MC = 0	MC > 0	Male	Female	Purebreed	Mixed Breed
All cases (*n* = 30) (*n*/%)	14 (46.7)	7 (23.3)	9 (30.0)	3 (10.0)	27 (90.0)	25 (83.3)	5 (16.7)	17 (56.7)	13 (43.3)	19 (63.3)	11 (36.7)
OC0 (*n* = 20) (*n*/%)	6 (30.0)	6 (30.0)	8 (40.0)	1 (5.0)	19 (95.0)	19 (95.0)	1 (5.0)	11 (55.0)	9 (45.0)	14 (70.0)	6 (30.0)
OC1 (*n* = 10) (*n*/%)	8 (80.0)	1 (10.0)	1 (10.0)	2 (20.0)	8 (80.0)	6 (60.0)	4 (40.0)	6 (60.0)	4 (40.0)	5 (50.0)	5 (50.0)

Abbreviations: OC0, dogs with postsurgical resolution of disease; OC1, dogs that died or were euthanized as a result of mast cell tumor disease progression.

**Table 2.** Descriptive statistics for continuous variables of patient demographics and tumor characteristics for all cases and by outcome status.

	Mitotic Count (As Continuous Variable)						Age					
	Mean	SD	Q1	Median	Q3	Range	Mean	SD	Q1	Median	Q3	Range
All cases (*n* = 30)	1.5	4.9	0.0	0.0	0.0	0–24	8.7	3.5	6.0	9.0	10.0	2–20
OC0 (*n* = 20)	0.1	0.4	0.0	0.0	0.0	0–2	7.3	2.7	5.0	7.5	9.0	2–12
OC1 (*n* = 10)	4.4	7.9	0.0	0.0	5.0	0–24	11.4	3.4	9.3	10.5	12.0	8–20

Abbreviations: OC0, dogs with postsurgical resolution of disease; OC1, dogs that died or were euthanized as a result of mast cell tumor disease progression; *n*, number of cases; SD, standard deviation; Q, quartile.

Regarding sex and breed, 17 were males (57%) and 13 were females (43%) (Table 1) (data regarding neutering status were not available nor collected), including 11 mixed breed dogs, 6 Labrador retrievers, 2 Bouvier Bernois, 2 French bulldogs, 2 Shar-peis, 1 boxers, 1 poodle, 1 Chihuahua, 1 German shepherd, 1 Serra de Aires dog, and 1 Doberman pinscher (Supplemental Table S1). Owing to the large breed dispersion, the breeds were additionally dichotomized and categorized as purebred (19 dogs, 63%) and mixed breed (11 dogs, 37%) (Table 1). The mean age at surgical excision was 8.7 years (SD = 3.5) (Table 2), and the follow-up period ranged from 2 to 24 months.

In the analysis of outcome groups, the OC0 group included 20 dogs that were alive at the end of this study and had no signs of local or distant recurrence; however, 1 dog developed melanoma with nasal infiltration. The OC1 group included 10 dogs that died due to csMCT-related disease, 6 of which were euthanized. These dogs included 3 cases with histologically confirmed lymph node metastasis at the time of diagnosis, 6 cases of local recurrence (2 confirmed by histology) in which 2 had regional lymphadenomegaly, and 2 cases of presumptive distant visceral metastasis detected by sonography. When lacking histologic diagnosis, presumptive local recurrence and distant metastasis were based on the regrowth of a mass or visceral sonographic alterations.

Regarding the growth pattern, multinucleated cells, and MC, OC0 cases included 6 infiltrative, 6 combined, and 8 circumscribed tumors (Table 1). Only 1 tumor, with a circumscribed pattern, had an MC of 2 and multinucleated cells; all other cases had MCs of 0 and no multinucleated cells. The 10 OC1 cases included 8 infiltrative, 1 combined, and 1 circumscribed tumor (Table 1). Two of the 3 cases with multinucleation died during the study. Five tumors were in the “MC > 0” group (Table 1), with 4 deaths associated with MCT disease progression. All had an infiltrative pattern, and only 1 had multinucleated cells.

In terms of sex and breed, the OC0 group included 11 males and 9 females, 14 purebreed and 6 mixed breed dogs (Table 1), while the OC1 included 6 males and 4 females, 5 of which were purebred and 5 were mixed breed dogs (Table 1).

### $\bar{v_{v}}$ Reproducibility

**Figure 2.**
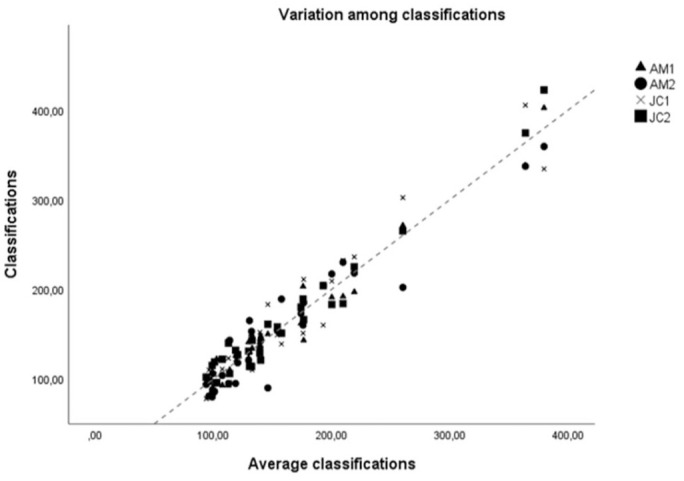
Comparison between each individual measurement (*y*-axis) and the average of the 4 measurements (*x*-axis). Each shape is one measurement. AM1, Ana Macara first measurement; AM2, Ana Macara second measurement 2; JC1, José Catarino first measurement 1; JC2, José Catarino second measurement.

For each tumor, $\bar{v_{v}}$ values were independently measured by 2 observers, in duplicate, totaling 4 analyses per tumor, each taking approximately 10–15 minutes (Table 3). Friedman’s test revealed no statistically significant differences when comparing measurements (χ^2^_*r*_ = 2.11, *P* = .55) and when comparing each measurement with the average of the 4 measurements (χ^2^_*r*_ = 3.15, *P* = .53) (Fig. 2). The concordance correlation coefficient was used to assess the agreement between each pair of measurements, with most coefficients consistently above 0.9, indicating excellent correlation between measurement sets (Table 4). In addition, the concordance correlation coefficient was employed to evaluate the agreement between each set of measurements and the average of the 4 measurements per sample, consistently yielding coefficients above .95 (Table 5). Consequently, the average was utilized as a representative value for $\bar{v_{v}}$ .

**Table 3.** Descriptive statistics for mean nuclear volumes (µm^3^) for each observer.

	*n*	Mean	SD	Q1	Median	Q3	Min.	Max.
C1	30	158.8	71.2	117.5	142.0	183.7	90.0	402.0
C2		155.9	69.0	98.0	143.5	188.0	80.0	359.0
C3		160.4	76.8	111.0	132.5	182.5	78.0	405.0
C4		161.9	76.3	116.0	136.0	182.2	86.0	422.0

Abbreviations: *n*, number of measurements per observer; SD, standard deviation; Q, quartile; C1, C2—observer 1; C3, C4—observer 2.

**Table 4.** Mean nuclear volume concordance correlation coefficient between the 4 independent analyses.

Pair	rc	95% CI
C1 vs C2	0.92	0.84, 0.96
C1 vs C3	0.90	0.81, 0.95
C1 vs C4	0.96	0.92, 0.98
C2 vs C3	0.86	0.73, 0.93
C2 vs C4	0.89	0.79, 0.95
C3 vs C4	0.93	0.86, 0.97

Abbreviations: C1, C2, C3, and C4—Each set of 30 measurements; C1, C2—observer 1; C3, C4—observer 2; ρc—Lin’s concordance correlation coefficient; 95% CI, 95% confidence interval.

**Table 5.** Mean nuclear volume concordance correlation coefficient between each independent analysis and the average.

Pair	ρc	95% CI
C1 vs average 4 measurements	0.97	0.95, 0.99
C2 vs average 4 measurements	0.95	0.90, 0.98
C3 vs average 4 measurements	0.96	0.91, 0.98
C4 vs average 4 measurements	0.98	0.96, 0.99

Abbreviations: C1, C2, C3, and C4—Each set of 30 measurements; C1, C2—observer 1; C3, C4—observer 2; ρc—Lin’s concordance correlation coefficient; 95% CI, 95% confidence interval.

### Prognostic Value of $\bar{v_{v}}$

The infiltrative pattern was more common in the poor outcome group, but a multivariable logistic regression considering age and sex was performed to evaluate its capability of predicting death, and no statistically significant differences were found between patterns (*P* > .05). The MC as a continuous variable was not associated with outcome in the univariate logistic regression (*P* = .20), but the dichotomized approach showed a significant association between cases with MC > 0 and MCT-related deaths (*P =* .04; β = 2.54; OR = 12.67; 95% CI = 1.18, 136.28; Table 6).

**Table 6.** Logistic regression between dichotomized mitotic count and outcome.

Variable	β	SE	Wald	*P*	OR	95% CI
Mitotic count dichotomized	2.54	1.21	4.39	.04*	12.67	1.18, 136.28
Outcome	–1.39	1.19	1.54	.21		

Abbreviations: SE, standard error; OR, odds ratio; 95% CI, 95% confidence interval.

The value in bold and * indicates statistical significance (*P* < .05).

Breed-group analysis for outcome status showed an increased number of OC1 cases in the mixed breed dogs (*n* = 5) and Labrador retrievers (*n* = 4) (Supplemental Table S1). A univariate logistic regression analysis comparing mixed breed versus purebred dogs showed no statistically significant differences in outcome status (*P* = .29). Owing to the large number of categories and asymmetric distribution, breed was only included in multivariable models when dichotomized. A univariate logistic regression analysis on sex revealed no statistically significant differences in outcome status (*P* > .05). When comparing age and outcome status, differences were observed between OC0 (7.3; SD = 2.7) and OC1 (11.4; SD = 3.4) (Table 2), and a univariate logistic regression showed that older animals have increased probability of a poorer outcome (*P* = .02; β = .61; OR = 1.84; 95% CI = 1.12, 3.03; Table 7).

**Table 7.** Logistic regression between age and outcome.

Variable	β	SE	Wald	*P*	OR	95% CI
Age	0.61	0.25	5.81	.02*	1.84	1.12, 3.03
Outcome	–6.33	2.48	6.53	.01		

Abbreviations: SE, standard error; OR, odds ratio; 95% CI, 95% confidence interval.

The value in bold and * indicates statistical significance (*P* < .05).

Correlations between clinicopathological characteristics and $\bar{v_{v}}$ were statistically significant only for the dichotomized MC (ρ = 0.79; *P* < .01) and age (*r* = 0.46; *P* < .05). Results for MC indicated multicollinearity, leading to its exclusion from multivariable analysis. The Kruskal-Wallis test revealed no statistically significant differences between the median $\bar{v_{v}}$ values across different histological patterns (*P* > .05). A univariate linear regression analysis showed that $\bar{v_{v}}$ was significantly associated with age (*P* = .02; β = 9.28; 95% CI = 2.35, 16.22; Table 8). $\bar{v_{v}}\;$ ranged from 94 to 200 µm^3^ in the OC0 group and 101 to 379.3 µm^3^ in the OC1 group (Table 9). The probability of $\bar{v_{v}}$ being associated with death was analyzed and was statistically significant on univariate logistic regression analysis (*P* = .01; β = .04 OR = 1.04; 95% CI = 1.01, 1.07; McFadden’s pseudo-R^2^ = .38; Table 10). To further analyze this association, backward multivariable regression models were performed. The goodness-of-fit of the logistic regression model was evaluated using the Hosmer-Lemeshow test, which yielded a *P* > .05 indicating an acceptable fit of the data. $\bar{\;v_{v}}$ was considered statistically significant and was associated with outcome in 2 models with the one including $\;\bar{\;v_{v}}$ and age showing the best performance (*P* = .045; β = .04; OR = 1.04; 95% CI = 1.00, 1.07; McFadden’s pseudo-R^2^ = .50; Table 10).

**Table 8.** Linear regression between age and mean nuclear volume.

Variable	β	SE	*P*	95% CI	Range
Age	9.28	3.37	.02*	2.35, 16.22	94.0–200.0
MNV	78.77	31.60	.01	14.05, 143.49	101.0–379.3

Abbreviations: MNV, mean nuclear volume; SE, standard error; 95% CI, 95% confidence interval.

The value in bold and * indicates statistical significance (*P* < .05).

**Table 9.** Mean nuclear volumes (µm^3^) within clinical outcomes of 30 scMCTs.

Outcome	*N*	Mean	SD	Q1	Median	Q3	Range
OC0	20	127.3	29.8	105.1	120.5	136.0	94.0–200.0
OC1	10	220.4	90.2	174.4	192.9	249.8	101.0–379.3

Abbreviations: OC0, dogs with postsurgical resolution of disease; OC1, dogs that died or were euthanized as a result of mast cell tumor disease progression; n, number of cases; scMCTs, subcutaneous canine mast cell tumors; SD, standard deviation; Q, quartile.

**Table 10.** Logistic regression models between mean nuclear volume and outcome.

	Variable	β	SE	Wald	*P*	OR	95% CI	McFadden’s Pseudo-R^2^
Univariate	MNV	0.04	0.01	6.41	.011*	1.04	1.01, 1.07	.38
	Outcome	–6.53	2.33	7.83	.005	n/a	n/a	
Multivariate	MNV	0.03	0.02	4.03	.045*	1.03	1.00, 1.06	.50
	Age	0.05	0.33	2.72	.099	1.72	0.90, 3.28	
	Outcome	–10.60	4.32	6.03	.014	n/a	n/a	

Abbreviations: MNV, mean nuclear volume; SE, standard error; OR, odds ratio; 95% CI, 95% confidence interval.

The values in bold and * indicate statistical significance (*P* < .05).

## Discussion

Published grading schemes are less clearly defined for csMCTs when compared with ccMCTs.^8,9,37^ Thompson et al^34^ previously reported that an infiltrative growth pattern, presence of multinucleation, and MCs were found to be negatively associated with survival. These features are prone to interobserver variability and bias. In that study, the infiltrative pattern showed statistically significant differences for predicting survival only when compared with the circumscribed pattern but not when compared with the combined pattern. In fact, recent studies^8,14^ have overlooked the risk factors reported by Thompson et al^[Bibr bibr8-03009858251315094] 34^ when classifying csMCTs and applied instead the 2-tier grading scheme.

Results of our study indicate that an infiltrative growth pattern tends to be more common in dogs with poor outcome and with a lower survival probability; however, no statistical significance was identified in this study. This finding is similar to previous studies, in which the pattern was also not associated with overall survival.^9,14^ MC and multinucleation are criteria associated with a poorer prognosis in several grading schemes, even though different cut-off values are used in the different schemes. Statistically significant associations were identified in the current study only when MC was dichotomized into MC = 0 and MC > 0. While most dogs in the MC > 0 group died, 6 deaths also occurred in the MC = 0 group. Regarding multinucleation, only 3 dogs had tumors containing multinucleated cells, and of these, 2 died. However, small sample size (*n* = 3) limits the applicability of this finding, making it difficult to draw definitive conclusions about the true significance of multinucleation in predicting outcomes.

The MC has been under heavy scrutiny recently. Determining MCs is subjective, time-consuming, and poorly standardized, which leads to high interobserver variation. Multiple cut-off values have been used, with variable performances, and the assessed area and the terminology (MC vs mitotic index) have not always been standardized making it difficult to compare and to apply thresholds.^17,26,27,34^ In addition, standardization of the morphologic characteristics of the mitotic figures and distinguishing atypical mitotic figures from mitotic-like figures also need to be defined in order to improve MC consistency, reproducibility, and accuracy obtained from both manual (on a routine glass slide approach or whole-slide imaging) and computational pathology approaches.^1,12^ One should always be aware that there is no direct mathematical relationship between the number of events (mitotic figures) in a single histological section and the number of the same events in 3D tissue space, meaning that independently of how good an algorithm can be at detecting mitotic figures, that number can be different when the subsequent histological section of the same paraffin block is evaluated.^5^ The same types of criticism can be applied to identifying multinucleated cells. All these issues signal a need of unifying criteria capable of better predicting outcome.

Mixed breed dogs and Labrador retrievers were more common in the poor outcome group. Labrador retrievers are recognized as being predisposed to MCTs,^2,13,32,33^ a trend also evident in Portugal, where a recent study identified them as the most affected breed.^18^ The high number of Labrador dogs with a poor outcome was interpreted as consequence of this overrepresentation. However, this association needs confirmation through a more comprehensive study with a larger number of animals.

In this study, a correlation between age and poor outcome was found. The literature describes age as a factor impacting both the probability of a dog developing an MCT^26^ and the likelihood of these tumors being of a higher grade,^20,30,31^ which, in theory, is associated with a poorer prognosis. Advanced age may also play a role in the decision to euthanize, which could affect the outcome data. It is also noteworthy that the groups have a limited number of animals, and upon individual analysis, there was one 20-year-old dog in the poor outcome group, which considering the small sample size, might also contribute to explaining this association.

The point-sampled intercept method allowed the measurement of $\bar{v_{v}}$ in 30 csMCTs with high intraobserver and interobserver reproducibility, taking approximately 10–15 minutes per tumor. This observation corroborates this method as reproducible, a finding which has also been documented for other tumors, such as human melanoma,^6^ human prostatic cancer,^24^ and recently ccMCTs.^7^ Casanova’s study on ccMCTs revealed $\bar{v_{v}}$ values between 87.1 and 214.2 µm^3^, which align closely with the findings observed in the current study. That study also evaluated the correspondence between $\bar{v_{v}}$ and the 2- and 3-tier grading schemes, with the identification of cut-offs for the different grades.^7^ In the present investigation, however, $\bar{v_{v}}$ showed no direct association with csMCTs patterns. This disparity observed between $\bar{v_{v}}$ and reported risk factors might stem from the subjective nature of qualitatively assessing histopathological characteristics, which could be exacerbated by the sample size of this study, and may have negatively impacted the performance of these parameters. However, these results do not dismiss the ability of tumors to exhibit a histopathological profile based on their biological behavior because tumors in the OC1 group with an infiltrative pattern tended toward higher $\bar{v_{v}}$ values. Employing a categorization system distinguishing between noninfiltrative and infiltrative patterns could potentially resolve the ambiguity surrounding this parameter. The results regarding histological patterns should be further explored in a study with a larger caseload.

An association between MC and mean nuclear volume was observed, but their strong correlation prevented its inclusion in the multivariable analysis. The correlation between MC and $\bar{v_{v}}$ is predictable, as increased mitotic activity is typically found in more aggressive tumors that also exhibit karyomegaly and noticeable nuclear pleomorphism. The measurement of $\bar{v_{v}}$ inherently considers these features, which can possibly explain its association with MC.

A statistically significant association between $\bar{v_{v}}$ and outcome was found in this study. The OC0 group had lower $\bar{v_{v}}$ compared with those with a poor outcome and this association was also significant on multivariable analysis. This association is consistent with prior studies linking higher $\bar{v_{v}}\;$ to tumors with a worse prognosis^29^ or tumor progression.^24^ As $\bar{v_{v}}$ is a quantitative measurement, reflecting nuclear size and pleomorphism, these results align with the recent consensus advocating the use of the 2-tier grading system, which uses nuclear pleomorphism as a factor, for MCT classification.^37^

In conclusion, this study indicates $\bar{v_{v}}$ as a potential prognostic factor to be considered when trying to predict outcome for csMCTs, while also emphasizing its ease of measurement and high reproducibility. Further validation of these findings through additional studies with larger sample sizes is recommended. A larger cohort study would also be beneficial for determining accurate cut-off $\bar{v_{v}}$ values.

## Supplemental Material

sj-docx-1-vet-10.1177_03009858251315094 – Supplemental material for Stereological estimation of mean nuclear volume as a prognostic factor in canine subcutaneous mast cell tumors Supplemental material, sj-docx-1-vet-10.1177_03009858251315094 for Stereological estimation of mean nuclear volume as a prognostic factor in canine subcutaneous mast cell tumors by José Catarino, Ana Macara, André Barros, David Ramilo, Filipa Coelho, Joana Santos and Pedro Faísca in Veterinary Pathology
